# Vietnamese Families' Strength and Resilience and Healthcare Professionals’ Role During the Pandemic

**DOI:** 10.1093/geroni/igab046.1383

**Published:** 2021-12-17

**Authors:** Christina Miyawaki, Minhui Liu, Kyriakos Markides

**Affiliations:** 1 University of Houston, Houston, Texas, United States; 2 Central South University, Changsha, Hunan, China (People's Republic); 3 University of Texas Medical Branch, Galveston, Texas, United States

## Abstract

Traumatic escape from Vietnam in 1975 brought 1.3 million Vietnamese refugees to the U.S. Today, Vietnamese are the largest Asian subethnic group in Houston, Texas (81,000+), making Houston the 3rd largest Vietnamese-populated city in the U.S. Despite these numbers, health research on Vietnamese population is limited. To address this gap, we developed the Vietnamese Aging and Care Survey and collected data on Vietnamese older adults (≥65 years) and their caregivers (N=199). The purpose of this study was to examine the association between caregivers’ caregiving characteristics and care recipients’ mental health (N=58 dyads). Descriptive statistics and logistic regression models were used. Caregivers were on average 53 years-old, Vietnam-born (97%), and working (66%). The majority (84%) lived with their care recipients and provided care for 20+ hours/week (69%) in good/excellent health (76%). Care recipients were on average 75 years-old, Vietnam-born (100%) in fair/poor health (81%). Regression results showed stressed caregivers with more-depressed care recipients (OR=1.47, 95%CI:1.02, 2.13) but positive caregiving experiences (OR=0.85, 95%CI:0.74, 0.97) and burdened caregivers (OR=0.79, 95%CI:0.65, 0.96) with less-depressed care recipients. We found the association between stressed caregivers and depressed care recipients (Life Stress Paradigm), but care recipients becoming a “helpful company” reduces caregiver burden and care recipients’ depression (Social Exchange Theory). Vietnamese families live in multigenerational households within ethnic enclaves and remain a tightly-knit family unit showing resilience to their low socioeconomic status (≤25K, 91%). Leveraging a family as their strength, healthcare professionals should take a caregiver-care recipient dyad approach when planning COVID-19 pandemic interventions in Vietnamese communities.

